# Development of Amperometric Biosensors Based on Nanostructured Tyrosinase-Conducting Polymer Composite Electrodes

**DOI:** 10.3390/s130506759

**Published:** 2013-05-21

**Authors:** Stelian Lupu, Cecilia Lete, Paul Cătălin Balaure, Dan Ion Caval, Constantin Mihailciuc, Boris Lakard, Jean-Yves Hihn, Francisco Javier del Campo

**Affiliations:** 1 Department of Analytical Chemistry and Environmental Engineering, Faculty of Applied Chemistry and Materials Science, University Politehnica of Bucharest, Polizu Gheorghe 1-5, Bucharest 011061, Romania; E-Mail: dan_caval@yahoo.com; 2 Laboratory of Electrochemistry, Institute of Physical Chemistry “Ilie Murgulescu” of the Romanian Academy, Splaiul Independentei 202, Bucharest 060021, Romania; E-Mail: clete@chimfiz.icf.ro; 3 Department of Organic Chemistry, Faculty of Applied Chemistry and Materials Science, University Politehnica of Bucharest, Polizu Gheorghe 1-5, Bucharest 011061, Romania; E-Mail: pbalaure@gmail.com; 4 Department of Physical Chemistry, Faculty of Chemistry, University of Bucharest, Bld. Regina Elisabeta 4-12, Bucharest 030018, Romania; E-Mail: pc_mihailciuc@yahoo.com; 5 Institut UTINAM, CNRS-UMR 6213, Université de Franche-Comté, 16 route de Gray, Besançon Cedex 25030, France; E-Mails: blakard@univ-fcomte.fr (B.L.); jean-yves.hihn@univ-fcomte.fr (J.-Y.H.); 6 Instituto de Microelectrónica de Barcelona, IMB-CNM (CSIC), Campus Universidad Autónoma de Barcelona, Bellaterra 08193, Barcelona, Spain; E-Mail: javier.delcampo@csic.es

**Keywords:** amperometric biosensor, microelectrode arrays, polythiophene enzyme biosensor, tyrosinase-polythiophene composite, alternating waveform polymerization potential, dopamine, catechol

## Abstract

Bio-composite coatings consisting of poly(3,4-ethylenedioxythiophene) (PEDOT) and tyrosinase (Ty) were successfully electrodeposited on conventional size gold (Au) disk electrodes and microelectrode arrays using sinusoidal voltages. Electrochemical polymerization of the corresponding monomer was carried out in the presence of various Ty amounts in aqueous buffered solutions. The bio-composite coatings prepared using sinusoidal voltages and potentiostatic electrodeposition methods were compared in terms of morphology, electrochemical properties, and biocatalytic activity towards various analytes. The amperometric biosensors were tested in dopamine (DA) and catechol (CT) electroanalysis in aqueous buffered solutions. The analytical performance of the developed biosensors was investigated in terms of linear response range, detection limit, sensitivity, and repeatability. A semi-quantitative multi-analyte procedure for simultaneous determination of DA and CT was developed. The amperometric biosensor prepared using sinusoidal voltages showed much better analytical performance. The Au disk biosensor obtained by 50 mV alternating voltage amplitude displayed a linear response for DA concentrations ranging from 10 to 300 μM, with a detection limit of 4.18 μM.

## 1. Introduction

**Scheme 1.**
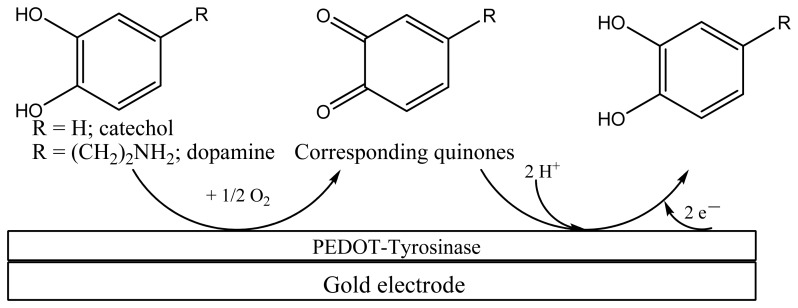
Generalized scheme of electrochemical biosensors based on immobilized tyrosinase.

New analytical devices for monitoring the quality of the environment and (bio)industrial processes are currently at the forefront of research. Electrochemical sensors and biosensors have rapidly emerged in recent decades as sensitive, reliable, and robust analytical devices [1–3]. Use of conducting polymer (CPs)-based (bio)composite materials as sensing elements in biosensor technology has proved to be a viable tool for achieving enhanced analytical performances [4–7]. The most commonly used CPs are polypyrrole (PPy), polyaniline (PANI), and various polythiophene (PTh) derivatives. Poly(3,4-ethylenedioxythiophene) (PEDOT) is one of the most commonly employed CPs, as a result of its excellent electrochemical and optical properties. This polymer can be simply obtained by anodic oxidation of the relevant monomer, even in aqueous solutions, in the presence of various anionic counterions. Composite with enzymes are also obtained by inclusion of the enzyme, under anionic form, during the polymerization-electrodeposition process [8–15]. Tyrosinase (Ty, polyphenol oxidase, E.C. 1.14.18.1) is a copper-containing enzyme that, in the presence of oxygen, catalyzes hydroxylation and oxidation of mono-phenols to *o*-quinones (monophenolase activity), and oxidation of *o*-diphenols to *o*-quinones (diphenolase activity) [16]. The resulting o-quinone can be reduced at the electrode surface at mild potentials, yielding *o*-diphenol (see Scheme 1). This is actually the functioning principle of several Ty-based amperometric biosensors [17–21]. The key issue of Ty-based amperometric biosensors lies in their low operational stability, due to loss of enzymatic activity following the enzyme immobilization on the transducer surface. Co-immobilization of Ty during electrochemical polymerization leading to PPy [22,23], PANI [24], PTh [25], and relevant copolymers [26] provides encouraging results in solving this issue. In particular, immobilization of Ty into PEDOT coating via potentiostatic methods has been addressed in several works [27–29]. Therefore, we have focused our attention on the development of a new, most effective immobilization method for Ty-based amperometric biosensor construction. This method consists in the use of sinusoidal voltages of various amplitudes superimposed on a dc potential. We recently demonstrated that PEDOT coatings prepared via sinusoidal voltages exhibit higher porosity and roughness, which can be suitable for enzyme immobilization [30]. Consequently, this method was further developed for Ty-coimmobilization in PEDOT coating during electrochemical polymerization of the corresponding monomer in aqueous buffered solutions.

The PEDOT-Ty bio-composite materials were characterized by infrared reflection absorption spectroscopy (IRRAS), atomic force microscopy (AFM), and scanning electron microscopy (SEM), to investigate their chemical structure and morphology. The optimized biosensor was then used in electroanalysis of dopamine (DA) and catechol (CT) in aqueous buffered solutions. A multi-analyte detection protocol, based on a bipotentiostatic technique, and the intrinsic properties of the Au microelectrode array, was also developed. The linear response range, sensitivity, repeatability, and stability were finally investigated.

## 2. Experimental Section

### 2.1. Chemicals

All chemicals, *i.e.*, tyrosinase (E.C. 1.14.18.1, from mushroom, 3,610 units/mg solid, Sigma, St. Louis, MO, USA), dopamine hydrochloride (Fluka, Steinheim, Germany), catechol (Fluka), Na_4_[Fe(CN)_6_] × 10 H_2_O (Riedel-de-Haën, Seelze, Germany), K_3_[Fe(CN)_6_] (Sigma-Aldrich, Steinheim, Germany), LiClO_4_ (Sigma-Aldrich, Steinheim, Germany), KCl (Riedel-de-Haën), KH_2_PO_4_ (Riedel-de-Haën), K_2_HPO_4_ × 3 H_2_O (Merck, Darmstadt, Germany), and 3,4-ethylenedioxythiophene (EDOT, Aldrich) were used without any further purification. EDOT was used for electrochemical preparation of the corresponding polymer. Double distilled water was used to prepare aqueous solutions.

### 2.2. Electrochemical Measurements

Electrochemical experiments were carried out at room temperature with an Autolab potentiostat/galvanostat 302N (Ecochemie, Utrecht, The Netherlands) coupled to a PC running the GPES software, using a single-compartment three electrode cell. The working electrode was a 2 mm diameter gold disk. Two microelectrode arrays were alternatively used as the working electrodes, comprising 816 microelectrodes with a 10 μm radius. Arranged in a 24 × 34 square lattice, they exhibit a center-to-center separation of 100 μm along the x- and y-axes. These microelectrode arrays were fabricated on silicon chips using standard microfabrication techniques, as described in previous works [31]. An Ag/AgCl/KCl (3M) electrode was used as the reference electrode (Metrohm, Herisau, Switzerland), and platinum wire (Metrohm) as the auxiliary electrode, respectively. All electrochemical measurements were performed inside a Faraday cage. Before each electrochemical measurement, the surface of the working microelectrode array was electrochemically activated in 0.1 M KCl aqueous solution [30]. The cyclic voltammograms were recorded simultaneously at both microelectrode arrays from one chip, using the *bipot* module of the potentiostat, in the array mode. The impedance spectra were recorded with the FRA2 module in the 100 kHz–0.1 Hz frequency range, using a sinusoidal excitation signal (single sine) with an amplitude (ΔE_ac_) of 50 mV. The solutions for electrochemical polymerization and measurements were deaerated by bubbling high purity argon through, and an argon flow was maintained over the solution during the measurements, to minimise re-oxygenation. The analytical applications of the biosensor in amperometric detection of DA and CT were performed in air-saturated buffered aqueous solutions at various working potentials.

### 2.3. Preparation Procedures of PEDOT-Ty Films

PEDOT-Ty bio-composite coatings were electrodeposited onto the Au disk electrodes or the two separate microelectrode arrays present on a single chip, from a solution containing 0.05 M EDOT, various amounts of Ty ranging from 0.1 to 3 mg/mL, and a 0.1 M phosphate buffer (pH 7.5) using the following procedures:

(A) sinusoidal voltages at a frequency within the 100 kHz–0.1 Hz range, with an excitation amplitude (ΔE_ac_) of 50 and 100 mV, were applied at a fixed dc potential value of +0.95 V to one of the chip arrays. Deposition time was 270 s. The resulting modified electrode was referred to as Au/PEDOT-Ty-SV; reference is given for Au disk electrode (*2-mm*) or microelectrode arrays (*array*) in this acronym throughout the text;
(B) the PEDOT-Ty coating was deposited onto the second array of the same chip using the potentiostatic method, *i.e.*, polarizing the elements of the array at a fixed potential value of +0.95 V *vs.* Ag/AgCl for 270 s deposition time. The deposition time was in this case the same as that used in the electrochemical polymerization process performed via sinusoidal voltages. The resulting modified electrode was referred to as Au/PEDOT-Ty-CA. The bio-composite coatings were also electrodeposited using both procedures (A) and (B) on 2-mm gold disk electrode, to investigate the performance of the new preparation procedure. After deposition of PEDOT-Ty bio-composite coatings, the device was thoroughly washed with distilled water and subjected to further investigations in aqueous solutions by using the cyclic voltammetry (CV).

### 2.4. Surface Characterization

The surface characterization of the coatings was performed on gold layers deposited on silicon. 100 nm-thick gold layers were deposited by sputtering, onto a silicon wafer (100-oriented standard 4” silicon wafers from Siltronix (Archamps, France), B-doped, resistivity: 0.015 ± 0.005 Ω·cm, thickness: 250 ± 10 μm). PEDOT and PEDOT-Ty samples were investigated by InfraRed Reflection Absorption Spectroscopy (IRRAS) in reflection geometry at a grazing-incidence angle of 65°. The infrared spectrometer used was a Vertex 70 FT-IR spectrometer (Ettlingen, Germany) equipped with a DTGS detector. For each sample, 128 infrared scans were performed at a resolution of 4 cm^−1^.

Imaging of the surface topographies took place with a commercial atomic force microscope (AFM PicoSPM from Molecular Imaging, Ann Arbor, MI, USA), in contact mode with aluminum coated silicon tip. The Si rectangular AFM cantilever was 450 μm–long, with a stiffness of 0.27 N·m^−1^. Experiments were conducted in air and at room temperature. The morphological features of all coatings were studied using a high resolution SEM (Quanta 450 W, FEI, Eindhoven, The Netherlands) with an electron beam energy of 10 keV.

### 2.5. Analytical Protocols

Analytical detection measurements were taken in an air saturated aqueous buffer solution at T = 294 ± 1 K. The chronoamperometric responses were recorded in continuous mode during addition of the analyte, while stirring. The multi-analyte detection protocol consists in simultaneously polarizing both microelectrode arrays from one chip using the bipot module in *bipot* mode. The bipot module in the *array* mode was applied for comparison of the analytical performances of various coatings, *i.e.*, PEDOT and PEDOT-Ty prepared by potentiostatic method, and PEDOT-Ty prepared by sinusoidal voltages.

## 3. Results and Discussion

### 3.1. Surface Characterization of PEDOT-Ty Bio-Composite Coatings

**Figure 1.**
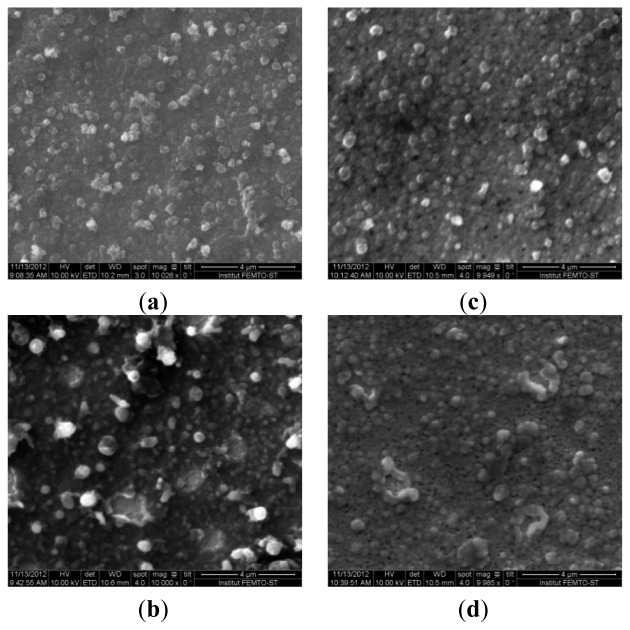
2D SEM image of (**a**) PEDOT and (**b**) PEDOT-Ty coating deposited by potentiostatic procedure; 2D SEM image of (**c**) PEDOT and (**d**) PEDOT-Ty coating deposited by sinusoidal voltage of 50 mV amplitude.

Optimization of the procedure for electrodeposition of the bio-composite coating was based on preliminary results concerning electrodeposition of pure PEDOT on both Au disk electrode and microelectrode arrays [30]. The porosity degree of the PEDOT coating was the key parameter used in the optimization of the preparation procedure: high porosity of the PEDOT matrix is beneficial for improved enzyme immobilization. The highest porosity was obtained with a sinusoidal voltage amplitude of 50 mV. This amplitude value was thus used in further studies on Ty co-immobilization. To investigate the morphology and chemical nature of the electrodeposited bio-composite coatings, Au layers deposited onto silicon wafer were used as substrates for sample preparation. Figure 1 shows the SEM images of PEDOT and PEDOT-Ty bio-composite coatings deposited by potentiostatic method and by sinusoidal voltage of 50 mV amplitude, respectively.

The morphology of the PEDOT matrix obtained by modulation of the applied potential is compared with that of the polymer coating grown under potentiostatic conditions (Figure 1(a,c), respectively). Its higher roughness reflects the modulation in the rate of polymer deposition and in parallel p-doping, with consequent inclusion of counterions from the solution. When the growth occurs in the presence of Ty, which is under anionic form, enzyme is also included as a counterion balancing the positive charges of polarons on the polymer. As a consequence, co-immobilization of Ty within the PEDOT matrix results; this seemingly makes the morphology of the surface (Figure 1(d)) much smoother than in the case of the Ty-PEDOT composite grown by using the potentiostatic method, whose typical SEM image is reported in Figure 1(b). The amount of immobilized Ty increases in the case of bio-composite coatings obtained via the sinusoidal voltage procedure, which is supported by the analytical performance of the prepared biosensor, as shown further on.

**Figure 2.**
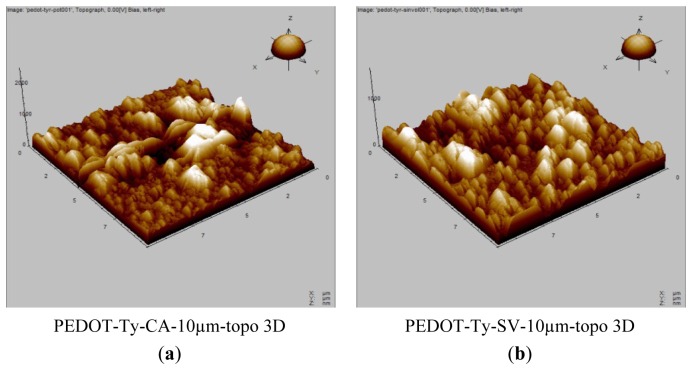
(**a**) 3D AFM image of PEDOT-Ty coating deposited by potentiostatic procedure (CA); (**b**) 3D AFM image of PEDOT-Ty coating deposited by sinusoidal voltage (SV) of 50 mV amplitude.

The morphology of the PEDOT-Ty bio-composite coatings prepared by both methods was also investigated using AFM. Figure 2 shows the 3D AFM images of PEDOT-Ty coatings obtained by either potentiostatic or sinusoidal voltage preparation procedure. Note that the PEDOT-Ty coating possesses a roughness, as expressed by RMS, of 700 nm, *i.e.*, less than the 900 nm value observed for the PEDOT-Ty coating obtained by the potentiostatic procedure. In the absence of enzyme, PEDOT films structure is porous. When an enzyme such as Ty is incorporated into this polymer structure, we hypothesize that the incorporation of enzyme also takes place inside the pores, leading to a lower RMS value in the presence than in the absence of enzyme. Consequently, the lower RMS value obtained when a sinusoidal voltage is applied could indicate that a higher incorporation of enzyme is obtained.

We must be aware of the fact that the microscopic and morphologic images are indicative, though not definitive in concluding about the higher number of Ty molecules in contact with the solution when an alternating voltage is superimposed to the continuous one. They are, however, in agreement with the indirect datum given by the higher effectiveness in biosensing of the composite obtained under sinusoidal polarization. Indeed, the homogeneous PEDOT-Ty coating prepared by sinusoidal voltages proved to exhibit superior analytical performance with respect to the same coating obtained via the potentiostatic procedure, as it will be shown below.

**Figure 3.**
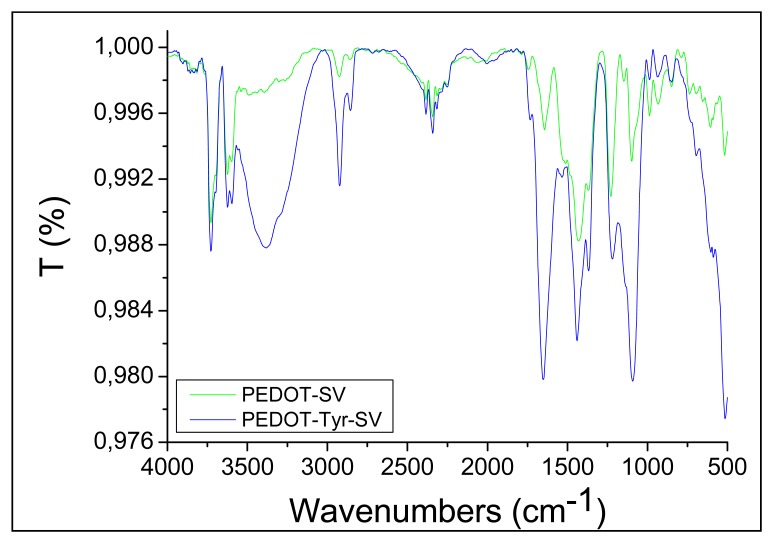
IRRAS spectra recorded for PEDOT and PEDOT-Ty coatings deposited using excitation amplitudes of 50 mV.

The chemical nature of the bio-composite coatings was investigated using the IRRAS technique. The IRRAS spectra of both PEDOT and PEDOT-Ty coatings are depicted in Figure 3.

The spectrum of PEDOT shows the characteristic peak vibrations at 1,490 cm^−1^ and 1,350 cm^−1^, which are attributed to stretching modes of C=C and C−C in the thiophene ring [23]. The highest peak at 1,700 cm^−1^, related to the doped state of PEDOT [32], showed the highest intensity for PEDOT samples prepared at 50 mV sinusoidal voltage amplitude. The higher doping level leads to higher density of positive charges on the PEDOT backbone, which are essential, in turn, to electrostatically immobilize the enzyme. Both PEDOT and PEDOT-Ty samples display a band at 3,700 cm^−1^ related to the O−H vibration mode due to the chemisorption of water and to the absence of drying before IR experiments. The band at 3,200–3,300 cm^−1^ observed in the spectrum of PEDOT-Ty coating is broad and can be attributed to amine and amide groups [33], and attests the successful immobilization of Ty within the PEDOT matrix. Therefore, IRRAS, AFM, and SEM measurements support the effectiveness of the sinusoidal voltage procedure in the preparation of PEDOT-Ty bio-composite coatings.

## 3.2. Influence of Sample Solution pH on Biosensor Response

**Figure 4.**
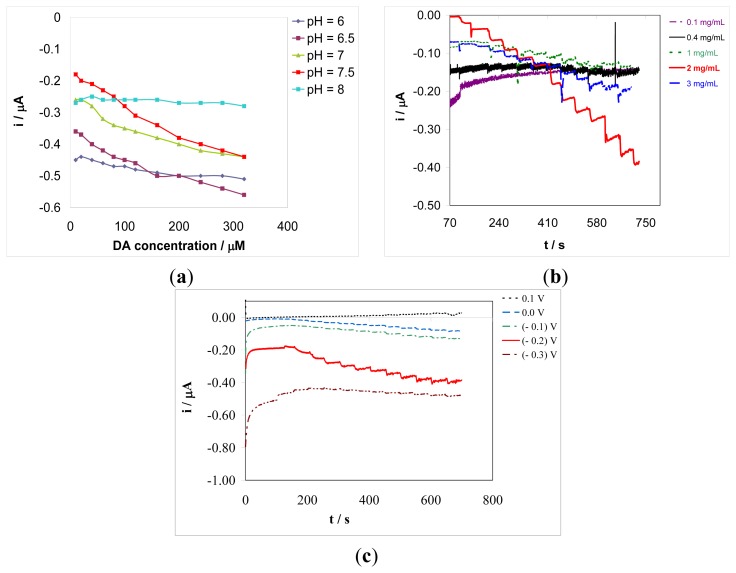
**(a)** Influence of the pH of solution on the gold disk electrode based biosensor response in aqueous solution containing a 0.1 M phosphate buffer and DA concentrations of 10, 20, 40, 60, 80, 100, 120, 160, 200, 240, 280, and 320 μM. Detection potential: −0.20 V *vs.* Ag/AgCl; (**b**) Influence of the enzyme concentration in the deposition solution on the gold disk electrode based biosensor response in aqueous solution containing a 0.1 M phosphate buffer of pH 7.5 and DA concentrations of 10, 20, 40, 60, 80, 100, 120, 160, 200, 240, 280, and 320 μM. Detection potential: −0.20 V *vs.* Ag/AgCl. (**c**) Influence of the detection potential in continuous amperometric mode under stirring conditions, on the gold disk electrode based biosensor response in aqueous solution containing a 0.1 M phosphate buffer of pH 7.5 and DA concentrations of 10, 20, 40, 60, 80, 100, 120, 160, 200, 240, 280, and 320 μM.

Influence of pH on the amperometric biosensor response was investigated within the pH range 6.0–8.0, with a 0.5 pH unit interval, in an air saturated aqueous buffer solution containing a fixed analyte concentration (see Figure 4(a)).

This study was conducted for all types of biosensors prepared either by superimposed sinusoidal voltage or by potentiostatic method, on both Au disk electrode and microelectrode arrays. The optimum pH value was found to be 7.5, which is close to the value of physiological samples. At such a pH the amperometric biosensor displays the most useful responses, linearly dependent in height on the analyte concentration over wide linear range.

### 3.3. Influence of Enzyme Concentration on Biosensor Response

The concentration of Ty in the deposition solution is a key operational parameter in the design of the amperometric biosensor. Therefore, the dependence of the biosensor response on Ty concentration was investigated within the range 0.1–3 mg/mL. This range was chosen for all types of biosensors prepared by sinusoidal voltage and potentiostatic procedure, on both Au disk electrode and microelectrode arrays. Biosensor responses were recorded in an air saturated aqueous buffer solution of pH 7.5 at various dopamine concentrations. Use of air saturated aqueous solution was dictated by the intrinsic functioning of Ty in amperometric biosensors and also fits the conditions in which the sensing system is actually employed. No net response was observed for enzyme concentration up to 0.4 mg/mL, while for Ty concentration up to 2 mg/mL the response increased significantly in height (see Figure 4(b)). However, enzyme concentrations higher than 2 mg/mL did not affect significantly the biosensor response. Therefore, 2 mg/mL was chosen as value of Ty concentration for biosensor construction by both preparation procedures, *i.e.*, potentiostatic and sinusoidal voltages.

### 3.4. Influence of the Working Electrode Potential on the Biosensor Response

Since the proposed biosensors are based on amperometric detection, the working potential is, of course, another key parameter in optimizing the analytical performance. In this context, the influence of the working potential on the sensitivity of the amperometric biosensor was investigated at various values (100 mV interval) between −0.30 and +0.10 V *vs.* Ag/AgCl. Figure 4(c) shows that, for the Au disk electrode biosensor, the best sensitivity, with respect to both more negative and less negative potentials, was achieved at a value of −0.20 V *vs.* Ag/AgCl. A large transient current is recorded at short times at the less positive potentials, that negatively affects the measure; it is related to the de-doping process of the PEDOT matrix. A compromise between a satisfactory sensitivity of measurements and a reduced transient current was reached at a working detection potential of −0.20 V, which was chosen for further analytical applications. The influence of the detection potential on the biosensor sensitivity was also examined on a microelectrode array amperometric biosensor: the best sensitivity was also in this case achieved for the detection potential value of −0.20 V *vs.* Ag/AgCl. Therefore, this potential value was used in further analytical applications.

### 3.5. Multianalyte Detection

**Figure 5.**
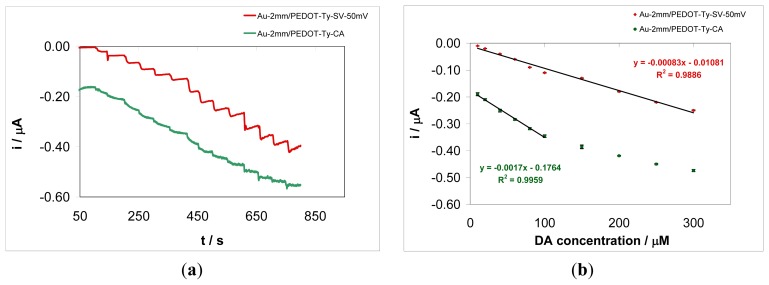
(**a**) Chronoamperometric responses recorded simultaneously at an Au-2mm/ PEDOT-Ty-SV-50mV modified electrode and an Au-2mm/PEDOT-Ty-CA modified electrode, respectively, in aqueous phosphate buffer solution at various dopamine concentrations: 10, 20, 40, 60, 80, 100, 150, 200, 250, 300, 400, 500, 600, and 700 μM. Working electrode potential: −0.20 V *vs.* Ag/AgCl; (**b**) Calibration plots obtained from the chronomperometric responses presented in Figure 5(a).

In this paper, a multianalyte detection protocol is developed. This protocol is based on the use of the *bipot* module of the potentiostat, in connection with the modified microelectrode (MEs) arrays, carrying out simultaneously measurements performed at an individual microelectrodes array from a chip or at two distinct modified Au disk electrodes, dipped in the sample solution. Alongside the benefit of the *bipot* module, the composition of the coatings can be tailored by preparing pure organic conducting polymer layers and bio-composite layers containing various amounts of enzyme on different electrode substrates. These approaches ensure enhancement of measurement selectivity and sensitivity. Selectivity can result only from the composition of coatings, while sensitivity is ensured by the mass transfer properties of the array and by the electrochemical detection mode. Measurements can be performed via two operating modes: (i) *array* mode—two electrodes, either conventional size electrodes or microelectrode arrays on a chip, are polarized simultaneously, and the electrode potential of the second electrode follows that of the first one; (ii) *bipot* mode—the potential of one electrode is scanned while the second electrode is held at a fixed potential value, which, in the case of interdigitated microelectrode bands, ensures detection of the electroactive species generated at the first electrode. The array mode may be used to achieve better selectivity of the overall biosensor response, consisting the responses of each individual sensing coating, both sensing layers being dipped in the same solution. Furthermore, this detection mode enables multianalyte detection, *i.e.*, monitoring several analytes present simultaneously in the sample. On the other hand, the *bipot* operation mode makes it possible to investigate the collection efficiency and redox cycling capability of the modified array based on interdigitated microelectrode bands. This operation mode enhances the analytical signal thanks to the redox cycling of electroactive species on the array, thus providing improved limits of detection and quantification. An example of the use of the *array* and *bipot* modes in developing multianalyte detection protocol will be given below. Firstly, the array mode is used in connection with 2-mm Au electrodes modified with bio-composite coatings, in order to show the enhanced analytical performance of biosensors prepared via sinusoidal voltages. Figure 5(a) shows the amperometric responses recorded simultaneously at an Au-2 mm/PEDOT-Ty-SV-50 mV modified electrode and an Au-2 mm/PEDOT-Ty-CA modified electrode, respectively, in aqueous phosphate buffer solution, at different DA concentrations. As it can be seen in this figure, the best analytical response was obtained for the bio-composite coating deposited via sinusoidal voltages using an amplitude of 50 mV. The response of this gold disk electrode based amperometric biosensor was linear up to a 300 μM DA concentration, according to the linear regression equation: i (nA) = −0.83 C_DA_ (μM)—10.81, with a squared correlation coefficient R^2^ of 0.9886 (r = 0.9943), as it can be seen in Figure 5(b). The sensitivity of this biosensor was 0.83 nA/μM, as obtained from the calibration plot. A detection limit (LD, 3 × *Sb*/*m*, where *Sb* is the standard deviation of the blank, n = 6, and *m* is the slope of the calibration curve) of 4.18 μM was obtained. The limit of quantification (LQ, 10 × *Sb*/*m*) for this amperometric biosensor was 13.95 μM. The corresponding LD and LQ for the Au-2mm/PEDOT-Ty-CA amperometric biosensor (for the linear response range from 10 to 100 μM DA) were 4.83 μM and 16.09 μM, respectively. The storage stability of the Au-2mm/PEDOT-Ty-CA biosensor was investigated by measuring its response for a 10 μM DA concentration after repeated storage at 4 °C in a phosphate buffer solution of pH 7.5. After 5 days' storage at 4 °C in a phosphate buffer solution, the biosensor response decreased by 35%. The repeatability of the biosensor responses was also investigated, for a series of nine successive measurements of 10 μM dopamine, and a value of 12.8% of the relative standard deviation was obtained. The storage stability of the Au-array/PEDOT-Ty-SV-50 mV biosensor was also investigated by using the same procedure as for the Au-2 mm/PEDOT-Ty-CA biosensor. After 5 days' storage at 4 °C in a phosphate buffer solution, the biosensor response decreased by 13%. The repeatability of the Au-array/PEDOT-Ty-SV-50 mV biosensor response was also investigated by measuring two times a 10 μM DA sample solution, and a value of 2.22% of the relative standard deviation was obtained. The Michaelis-Menten constant (K_m_) of the Au-2 mm/PEDOT-Ty-SV-50 mV biosensor was also evaluated from the corresponding Lineweaver-Burk plot. A K_m_ value of 1.84 mM was obtained, indicating a high affinity of immobilized Ty for DA. Although the biosensor prepared potentiostatically seems slightly more sensitive than the biosensor prepared using sinusoidal voltage (easy access to enzymes at the surface), the latter system shows a larger linear dependence between current and analyte concentration, and the signal in the absence of analyte is closer to zero, which facilitates sensor calibration. Therefore, the use of sinusoidal voltages has resulted in increased overall analytical performance of the new biosensors, attesting to the benefits of this new preparation procedure by providing a conducting polymer matrix with increased roughness and porosity. These features of the conducting polymer matrix have thus ensured enhanced immobilization of the enzyme, which results less prone to losses by desorption. Furthermore, the figures of merit of analytical performance corresponding to the amperometric biosensor prepared via sinusoidal voltages preparation procedure are superior in comparison with those related to the biosensor obtained by potentiostatic method and comparable with those [34–37] previously reported (see Table 1).

Bipotentiostatic measurements at microelectrode arrays allowed the simultaneous monitoring of DA and CT. One electrode of the chip was modified by a PEDOT-Ty bio-composite coating, while a pure PEDOT coating was electrodeposited on the second electrode of the chip. Deposition of these two coatings, with and without enzyme, aimed at highlighting the sensing abilities of each one of the two systems towards the DA and CT present simultaneously in the solution. Therefore, composition of the coatings was driven to provide information about selectivity of the overall response of the chip towards two analytes.

**Figure 6.**
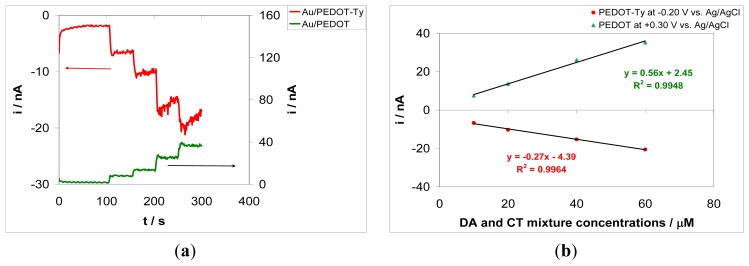
(**a**) Chronoamperometric responses recorded simultaneously at an Au-array/PEDOT-Ty-CA modified electrode, polarized at −0.20 V *vs.* Ag/AgCl, and an Au-array/PEDOT-CA modified electrode, polarized at +0.30 V *vs.* Ag/AgCl, in an aqueous phosphate buffer solution of pH 7.5 at various dopamine and catechol mixture concentrations: 10, 20, 40, 60, and 80 μM; (**b**) Calibration plots obtained from the chronomperometric responses presented in Figure 6(a).

Figure 6(a) shows the amperometric responses of each electrode recorded simultaneously in *bipot* mode in an aqueous phosphate buffer solution at pH 7.5, containing DA and CT at equal concentrations of 10, 20, 40, 60, and 80 μM. The PEDOT-Ty modified electrode was polarized at −0.20 V *vs.* Ag/AgCl, while the PEDOT modified electrode was polarized at +0.30 V *vs.* Ag/AgCl. The choice of these detection potential values is based on the bio-electrocatalytic and sensing properties of each coating, *i.e.*, the −0.20 V *vs.* Ag/AgCl value is set up for the PEDOT-Ty sensing layer according to the optimization of the biosensor response presented in Section 3.4, and the +0.30 V *vs.* Ag/AgCl value assures oxidation of DA only at the PEDOT coating, respectively. Therefore, the choice of these detection potential values is assumed to ensure improved sensitivity of chip overall response, while the coating composition affords better selectivity. In actual fact, the powerful properties of the arrays, the composition of the sensing layers, the detection potentials and the *bipot* mode all converge to the design of a multianalyte detection protocol.

The second electrode modified with PEDOT only was polarized at a potential value of +0.30 V *vs.* Ag/AgCl, *i.e.*, at a potential suitable to ensure oxidation of DA. However, this potential value is not positive enough to assure oxidation of CT. Therefore, at this electrode only the amperometric response of the PEDOT coating towards DA oxidation is observed. The PEDOT-Ty modified electrode is polarized at −0.20 V *vs.* Ag/AgCl, *i.e.*, a potential value that assures reduction of the quinone derivative produced in the enzymatic reaction with Ty. DA and CT compete for the Ty active centers, and there is a greater current increase after each addition of the DA-CT mixture for the PEDOT-Ty based biosensor with respect to the pure PEDOT based electrochemical sensor. The negative current obtained at the PEDOT-Ty modified electrode attests to proper functioning of the bio-composite coating. The relevant calibration plots are depicted in Figure 6(b). As it can be seen in this figure, there is a strong correlation between the analytical signal and the analyte concentrations. The amperometric responses were linear up to 80 μM DA-CT concentrations. Sensitivity of the PEDOT-Ty coating was 0.27 nA/μM, while for the PEDOT coating a sensitivity of 0.56 nA/μM was obtained. Sensitivity of the PEDOT coating is twice that of the PEDOT-Ty coating. This result points to the features of the arrays used in this work, mainly to the cycling capability. According to Scheme 1, the DA and CT molecules are reacting with Ty and the corresponding quinone derivatives are detected at the PEDOT-Ty modified electrode by their electrochemical reduction, which further supply the reacting molecules DA and CT as the products of this electrochemical reaction. The DA generated at the PEDOT-Ty modified electrode as the product of quinone derivative reduction is oxidized at the second electrode modified with PEDOT coating. The generated CT cannot be oxidized at the PEDOT modified electrode because of the low working potential, *i.e.*, +0.30 V *vs.* Ag/AgCl. Therefore, the sensitivity of the PEDOT coating is twice that of the PEDOT-Ty coating and this means that the recycling capability of the device is almost 100%. It is worth noting that each sensing layer was prepared in the same experimental conditions, to ensure the same thickness of the coating electrodeposited onto the electrode surface. Furthermore, the short distance between the electrodes reduces the ohmic drop between them when both electrodes are polarized simultaneously and provides a short diffusion path for generated DA molecules to reach the surface of PEDOT modified electrode. The response time of both sensing layers is less than 10 s, meaning that these amperometric (bio)sensors quickly equilibrate. We can assume that there is a substantial overlap of the analyte diffusion layer, because at the end of each steady state response, just before the addition of a new aliquot of analyte mixture, there is a slight decrease in current on the array-based biosensor. This behavior is typical of the geometry of the arrays used in this work, but should not be considered as a drawback, since the array behaves as a conventional size electrode with the same area but with an increased signal to noise ratio. The higher signal to noise ratio for arrays is a key parameter conditioning their choice as substrates for construction of these amperometric biosensors. These results show that by proper choice of composition of sensing layers and electrochemical detection parameters, two or more analytes can be simultaneously monitored. For instance, the PEDOT modified electrode may be polarized at a higher positive potential value that assure the oxidation of both DA and CT analytes. The quantitative determination of each analyte may be done by using multiple standard addition method following this protocol: the sample is spiked consecutively with DA only followed by addition of both DA and CT analytes in equal concentrations. The current increase recorded for DA only increment is subtracted from the current increase due to the addition of the mixture and this difference gives the CT concentration that contributes to the overall current. Further studies are currently in progress to design these operational parameters in order to use these amperometric biosensors for quantitative analysis of several analytes.

## 4. Conclusions

In this work, amperometric biosensors based on gold disk electrode and microelectrode arrays modified with PEDOT-Ty coatings and pure PEDOT were prepared and used in DA and CT electroanalysis. Biosensors were prepared via a classical method, chosen as a reference one, and via an original preparation procedure, respectively. The amperometric biosensors prepared using sinusoidal voltages exhibited enhanced analytical performance towards DA detection. The gold disk electrode based biosensor obtained by 50 mV amplitude displayed a linear response for DA concentrations ranging from 10 to 300 μM, with a detection limit of 4.18 μM. The microelectrode arrays based biosensor was used for simultaneous monitoring of DA and CT in *bipot* mode. A semi-quantitative analysis of two analytes present simultaneously in the solution using an amperometric biosensor and an electrochemical sensor was presented. These results show that the use of the bipot module in connection with arrays modified by various sensing layers allows us to set up protocols for simultaneous monitoring two analytes.

## Figures and Tables

**Table 1.** Analytical performances of tyrosinase based biosensors for dopamine detection.

**Linear Range** **(mol·L^−1^)**	**Detection Limit** **(mol·L^−1^)**	**Working Potential** **(V)**	**Reference**
5 × 10^−5^ to 2.5 × 10^−4^	2.5 × 10^−5^	−0.19 V *vs.* Ag/AgCl	20
up to 2 × 10^−4^	1 × 10^−4^	−0.20 V *vs.* SCE	27
N/A	2 × 10^−7^	−0.10 V *vs.* Ag/AgCl	34
2 × 10^−6^ to 1 × 10^−5^	9 × 10^−7^	Differential pulse voltammetry	35
5 × 10^−6^ to 23 × 10^−6^	0.52 × 10^−6^	−0.010 V *vs.* Ag/AgCl	36
1.2 × 10^−4^ to 3.6 × 10^−4^	39.60 × 10^−6^	−0.10 V *vs.* SCE	37
1 × 10^−5^ to 3 × 10^−4^	4.18 × 10^−6^	−0.20 V *vs.* Ag/AgCl	This work
